# Supplementary material for: Development and Psychometric Properties of a New Patient-Reported Outcome Instrument of Health-Related Quality of Life Specific to Patients with Gambling Disorder: The Gambling Quality of Life Scale (GQoLS)
Source: Int J Environ Res Public Health. 2022 Aug 30;19(17):10806. doi: 10.3390/ijerph191710806 (PMC9517863; doi:10.3390/ijerph191710806)
Supplement: Supplementary file 1 [file ijerph-19-10806-s001.zip › ijerph-1848285-supplementary.pdf]

SUPPLEMENTARY MATERIAL

Table S1. Construct validity of the GQoLS with EQ-5D and PGSI (n = 98)

	Mean (SD)	*r*
Mobility (n= 97)	1.06 (0.24)	-0.19
Self-care (n= 97)	1.02 (0.14)	0.06
Activity (n= 97)	1.27 (0.51)	-0.01
Pain (n= 97)	1.41 (0.60)	-0.06
Anxiety (n= 97)	2.08 (0.59)	0.26
Health state visual analog scale (VAS) (n = 96)	68.65 (20.50)	-0.19
PGSI	15.32 (5.00)	0.52

Table S2. ***Item-dimension correlation*** *Factor 1*

Item 14 (37)	*0.65*
Item 15 (39)	0.61
Item 16 (41)	0.73
Item 17 (44)	0.73
Item 18 (45)	0.68
Item 19 (46)	0.65
Item 20 (48)	0.52

Factor 2

Item 1 (2)	*0.61*
Item 2 (4)	0.65
Item 6 (10)	0.59
Item 7 (11)	0.70
Item 8 (16)	0.63

Item 9 (19)	0.71

Factor 3

Item 3 (6)	*0.50*
Item 4 (7)	0.64
Item 5 (8)	0.49

Factor 4

Item 10 (22)	*0.46*
Item 11 (27)	0.61
Item 21 (51)	0.52

Factor 5

Item 12 (31)	*0.59*
Item 13 (32)	0.59

Section S1 : Selection of items for GQoLS-BREF

1/ Selection of the two items of each dimension permitting the best internal consistency:

dim* 1: item 41 and item 44
dim 2: item 11 and item 19
dim 3: item 7 and item 8
dim 4: item 27 and item 51
dim 5: item 31 and item 32

2/ Selection of the two items of each dimension with the best load:

dim 1: item 41 and item 45
dim 2: item 11 and item 19
dim 3: item 7 and item 8
dim 4: item 27 and item 51
dim 5: item 31 and item 32

3/ Selection of the two items of each dimension with the best item-dimension correlations:_

dim 1: item 41 and item 44
dim 2: item 11 and item 19
dim 3: item 6 and item 7
dim 4: item 27 and item 51
dim 5: item 31 and item 32

*dim: dimension
